# Adverse health effects of experiencing food insecurity among Greenlandic school children

**DOI:** 10.3402/ijch.v72i0.20849

**Published:** 2013-08-05

**Authors:** Birgit Niclasen, Max Petzold, Christina W. Schnohr

**Affiliations:** 1 Greenlandic Branch, National Institute of Public Health, University of Southern Denmark, Copenhagen, Denmark; 2 Akademistatistik – Centre for Applied Biostatistics, University of Gothenburg, Gothenburg, Sweden; 3 Institute of Public Health, University of Copenhagen, Copenhagen, Denmark

**Keywords:** food insecurity, hungry to bed, adolescents, Greenland, HBSC, self-rated health, medicine use, symptoms

## Abstract

**Background:**

In vulnerable populations, food security in children has been found to be associated with negative health effects. Still, little is known about whether the negative health effects can be retrieved in children at the population level.

**Objective:**

To examine food insecurity reported by Greenlandic school children as a predictor for perceived health, physical symptoms and medicine use.

**Design:**

The study is based on the Greenlandic part of the Health Behavior in School-aged Children survey. The 2010 survey included 2,254 students corresponding to 40% of all Greenlandic school children in Grade 5 through 10. The participation rate in the participating schools was 65%. Food insecurity was measured as going to bed or to school hungry because there was no food at home.

**Results:**

Boys, the youngest children (11–12 year-olds), and children from low affluence homes were at increased risk for food insecurity. Poor or fair self-rated health, medicine use last month and physical symptoms during the last 6 months were all more frequent in children reporting food insecurity. Controlling for age, gender and family affluence odds ratio (OR) for self-rated health was 1.60 (95% confidence interval (CI 1.23–2.06) (p<0.001), for reporting physical symptoms 1.34 (95% CI 1.06–1.68) (p=0.01) and for medicine use 1.79 (95% CI 1.42–2.26) (p<0.001). Stratification on age groups suggested that children in different age groups experience different health consequences of food insecurity. The oldest children reported food insecurity less often and experienced less negative health effects compared to the younger children.

**Conclusions:**

All 3 measures of health were negatively associated to the occurrence of food insecurity in Greenlandic school children aged 11–17. Food security must be seen as a public health issue of concern, and policies should be enforced to prevent food poverty particularly among boys, younger school children and children from low affluence homes.

In the Arctic region, the issue of food insecurity is highly relevant. A concurrent finding within Arctic countries is that households with children and indigenous populations are at special risk (1–5). In 2004, Canadian Aboriginal households were 2.6 times more likely to report food insecurity compared to non-Aboriginal households (6). In Nunavut, 49% of households reported not having enough to eat “often” or “sometimes” during the year prior to the study, compared to 7% for overall Canadian households (1). A very high prevalence of food insecurity has been found in Inuit households with children. In 2007–2008, 31% of Inuit children in 16 Nunavut communities lived in moderate child food insecure homes, while another 25% lived in severe child food-insecure homes (3). In 2010, 27% of Alaskan households with children were food insecure compared to 19% of households in the United States (2).

Only a few studies have investigated the effects of food insecurity, food shortage or hunger in the general child population, while most have included children that are especially vulnerable to food insecurity due to concomitant social deprivation (7–11). Despite addressing a different set of correlates, taken together there is solid evidence among the international literature from vulnerable populations in rich countries that food insecurity in children is associated with a range of adverse effects on health, development, academic performance and behavioral or psychosocial problems. In Western countries, the health risks associated with food insecurity in children have been found to include, for example, greater odds of having health reported as “fair/poor” (vs. “excellent/good”), greater odds of hospitalisation since birth, increased rates of acute and chronic illness, lower health-related quality of life and experiencing more health complaints such as stomach aches and headaches (3,7,12–14). However, the relationships between food insecurity and children's health, behavior and development seem to vary according to the child's age, gender and ethnicity (7,13,15–17).

Most reports on food insecurity have been based on parental reports (1–5,9). However, it is important also to study associations with food insecurity as perceived by the children themselves. Children are valid reporters of their own experiences, and they experience food insecurity differently from adults both in content and context (18). Children report food insecurity based on their experiences of the relational and resource contexts they encounter. Their responses are based on their personal experiences, such as worries about parental stress and hardships, feelings of anger and helplessness, and cognitive vigilance to monitor the household food situation (18,19).

Since 2002, food insecurity in Greenlandic school children has been measured every 4 years as part of Greenland's participation in the international Health Behavior in School-aged Children (HBSC) survey. In both 2002 and 2006, Greenland had the highest proportion of children among the more than 40 participating countries reporting food insecurity (20). In both 2006 and 2010, 17% reported experiencing going to bed hungry “always” or “often”, while 69% in 2002, 59% in 2006 and 68% in 2010 reported it “never” happened to them (20–22). In Yukon, Canada, using HBSC 2010 data the highest proportion of children going to bed or to school hungry at least sometimes was found in rural boys in Grades 6 through 8 (43%) and the lowest in rural girls in Grades 9 through 10 (23%) (23), figures which are close to Greenlandic ones.

Given that the probable adverse health effects of food insecurity found in other Western countries can be applied to the population of Greenlandic children, a major public health concern is identified due to the high proportion of affected children.

The objective of this study was to examine food insecurity measured by reporting of going to bed or to school hungry because there was no food at home, as a predictor for perceived health, physical symptoms, and medicine use in Greenlandic school children.

## Methods

The study was based on data from the Greenlandic part of the Health Behavior in School-aged Children (HBSC) survey including children aged 11–17 (Grades 5 through 10). HBSC is a cross-national survey on child and adolescent health and health behavior. The survey is performed every 4 years and includes more than 40 countries and regions in Europe, North America and Israel (24). Greenland has taken part in the HBSC since 1994. Data from the 2010 survey included 2,254 students corresponding to 40% of all Greenlandic school children in the included grades. The participation rate in the participating schools was 65% (18).

Food insecurity was based on the answers to the following question: “Some young people go to school or to bed hungry because there is not enough food at home. How often does this happen to you?” with the response categories “always”; “often”; “sometimes”; and “never”.

Outcome variables were: self-rated health, “How would you say your health is?” (“excellent”, “good”, “fair”, or “poor”); physical symptoms, “In the last 6 months: how often have you had the following ….?” (i.e. headache, stomach ache and back ache – “about every day”, “more than once a week”, “about every week”; “about every month”, “rarely”, or “never”); and medicine use, “During the last month, have you taken any medicine or tablets for the following?” (i.e. headache, stomach ache, difficulties in getting to sleep, nervousness, and something else – “no”, “yes, once”, or “yes, more than once”).

Socio-demographic variables were selected based on findings in the literature (7,13,15–17). They included: age, gender, urban/rural habitation and household economic characteristics. Age was categorised as 11–12, 13–14 and 15–17 years-old respectively. Habitation was categorised into towns and villages according to the present legal registration. Household economic characteristics were measured by the Family Affluence Scale (FAS). FAS include 4 items on material assets in the family: own-bedroom (no=1/yes=2), family car (none=1/ one=2/2 or more=3), number of computers (none=1/ one=2/2 or more=3) and number of family holidays during the past year (none=1/one=2/2 or more=3). The 10-point FAS scale was trichotomised into high (7–9 points), medium (4–6) and low (0–3) including 38.3, 46.2 and 15.5% of children respectively. FAS has been shown to be a valid measure of the family's socioeconomic position (25). The latest validation study showed a high agreement between students’ reports and their parents’ reports on the 4 FAS-items (26). In Greenland, FAS has been validated against adult information on the same items and was found to correlate well to socioeconomic position in adults (19).

### Statistical procedures

Descriptive statistics were used to describe the data characteristics. The chi-squared test was used to evaluate differences in proportions of self-rated health, physical symptoms and medicine use. Then logistic regression models were built up in 3 steps. For the purpose of the analyses, dichotomous measures were constructed on the food poverty item (“always”or“often”/“sometimes”/“never”), on self-rated health (“fair” or “poor”/“excellent” or “good”), on physical symptoms (“about every day” or “more than once a week” or “about every week”/“about every month” or “rarely” or “never”) and on medicine use (“no”/“yes, once” or “yes, more than once”). Firstly, univariate analysis with good self-rated health, symptoms about every week or more often, and medicine use was performed. In the 2nd step, the analysis was repeated controlled for age, gender, type of habitation and FAS. In the 3rd step, the analyses were split on age groups.

As self-rated health, physical symptoms and medicine use were found somewhat interrelated they were not included in the same model.

## Results

Overall, 34.7% of the respondents reported to have suffered a headache, stomach ache or backache once or more than once within the past 6 months; 21.9% had used medicine the past month; and 18.4% reported fair or poor health.

### Proportions experiencing hunger due to food shortage at home

In total, 37.7% of boys and 26.5% of girls reported having experience going to school or to bed hungry because there was not enough food at home “sometimes”, “often” or “always” (Table I – result for “sometimes” not shown).

**Table I:** Proportions of children experiencing hunger due to food shortage at home shortage on self-rated health, physical symptoms, medicine use and socio-demographic factors

		Never (%)	Often or always (%)
Gender (p<0.001)	Boys Girls	62.3 73.5	16.7 9.3
Age (p=0.001)	11–12 years 13–14 years 15–17 years	65.3 67.3 72.3	17.0 12.4 9.4
FAS (p<0.001)	Low Medium High	60.5 71.4 76.5	14.8 12.0 9.8
Type of habitation (p<0.001)	Town Village	71.0 58.2	10.6 18.3
Self-rated health (p<0.001)	Good/excellent Fair/poor	70.0 60.3	12.6 13.9
Physical symptoms last 6 month (p=0.02)	About every week or more frequent Less than about every week	66.4 71.3	11.2 10.6
Medicine use last month (p<0.001)	Yes No	64.1 74.4	13.4 9.1

#### Health effects

*Self-rated health*. More children who had experienced food insecurity reported fair or poor health (39.7% vs. 30.0%) (p<0.001).

*Physical symptoms*. Food insecure children more frequently had experienced one or more physical symptom weekly or more often (33.6% compared to 28.7%) (p=0.02).

*Medicine use* More frequent in food-insecure children (25.6% compared to 35.9%) (p<0.001) (Table I – result for “sometimes” not shown).

*Socio-demographic factors*. Highly significant differences between experiencing food insecurity “never” and “sometimes”, “often” or “always” for all the socio-demographic factors investigated.

*Gender*. More boys than girls reported food insecurity “sometimes”, “often” or “always” (p<0.001).

*Age*. The younger children had experienced food insecurity more often than the older children (p<0.001).

*FAS and type of habitation*.The low FAS-group children and children in villages reported food insecurity most often (p<0.001 for both) (Table I).

### Multiple logistic regression

#### Self-rated health

Reporting poor self-rated health was significantly associated with experiencing food insecurity, with an odds ratio (OR) 1.54 (95% confidence interval (CI 1.21–1.95) (p<0.001). When adjusting for age, gender, FAS and type of habitation, the latter (i.e. habitation) was found to be insignificant and was excluded in subsequent analyses. Hence, in the final model controlling for age, gender and FAS, OR was attenuated to 1.60 (95% CI 1.23–2.06, p<0.001) (Table II). When splitting the analyses on age group, both crude rates and rates adjusted for gender and FAS in the 11–12 year-olds and the 13–14 year-olds showed significant associations, while no association to self-rated health was found in the oldest children (Table III).

**Table II:** Association between reported hunger (sometimes, often or always) and health outcomes [OR (95% CI)]

		Self-rated health (fair/poor)	Physical symptoms about every week or more frequent	Medicine use last month
Crude rates	Never Sometimes, often or always	1 1.54 (1.21–1.95) (p<0.001)	1 1.26 (1.01–1.56) (p=0.04)	1 1.62 (1.30–2.02) (p<0.001)
Adjusted for socio-demographic factors (p<0.001)	Never Sometimes, often or always	1 1.60 (1.23–2.06) (p<0.001)	1 1.34 (1.06–1.68) (p=0.01)	1 1.78 (1.42–2.26) (p<0.001)

**Table III:** Association between reported hunger (sometimes, often or always) and health outcomes split on age group [OR (95% CI)]

Age			Self-rated health (good/excellent)	Physical symptoms about every week or more frequent	Medicine use last month
11–12 years	Crude rates	Never	1	1	1
		Sometimes, often or always	1.73 (1.10–2.73) (p=0.02)	1.19 (0.79–1.79) (p=0.40)	1.84 (1.23–2.72) (p=0.003)
	Adjusted for socio-demographic factors (p<0.001)	Never	1	1	1
		Sometimes, often or always	2.02 (1.24–3.29) (p=0.005)	1.18 (0.78–1.80) (p=0.43)	2.09 (1.37–3.18) (p=0.001)
13–14 years	Crude rates	Never	1	1	1
		Sometimes, often or always	1.72 (1.16–2.54) (p=0.01)	1.39 (0.96–2.02) (p=0.08)	1.99 (1.31–2.77) (p=0.001)
	Adjusted for socio-demographic factors (p<0.001)	Never	1	1	1
		Sometimes, often or always	1.76 (1.16–2.66) (p=0.01)	1.53 (1.04–2.25) (p=0.03)	2.04 (1.37–3.03) (p<0.001)
15–17 years	Crude rates	Never	1	1	1
		Sometimes, often or always	1.32 (0.87–2.02) (p=0.19)	1.22 (0.84–1.76) (p=0.29)	1.32 (0.88–1.97) (p=0.17)
	Adjusted for socio-demographic factors (p<0.001)	Never	1	1	1
		Sometimes, often or always	1.10 (0.70–1.73) (p=0.68)	1.24 (0.83–1.84) (p=0.29)	1.53 (1.00–2.35) (p=0.05)

#### Physical symptoms

More children reporting food insecurity had had 1 or more physical symptoms during the past month with an OR of 1.34 (95% CI 1.06–1.68) (p=0.01). As seen for self-rated health, place of habitation was found to be insignificant when adjusting for age, gender, FAS and place of habitation, and was excluded in subsequent analyses. Controlling for the other socio-demographic factors, OR was 1.26 (95% CI 1.01–1.56) (p=0.04) (Table II). When splitting the analyses on age group only in the 13–14 year-olds significant association were found for crude rates and controlled for gender and FAS (Table III).

#### Medicine use

As for the other outcome variables place of habitation showed no significance and was excluded. In the case of both crude rates and rates adjusted for socio-demographic factors, highly significant number of children had used medicine 1 or more times in the last month, with an OR 1.62 (95% CI 1.30–2.02) (p<0.001) and OR 1.79 (95% CI 1.42–2.26) (p<0.001) respectively (Table II). Splitting the analyses on age group (Table III) still revealed that significantly more children who had experienced food insecurity also had taken medicine during the past month. This was found for both crude rates and rates adjusted for socio-demographic factors. The findings were most pronounced in the 2 younger age groups, and crude rate in the 15–17 year-olds was an exception from this pattern.

## Discussion

Food insecurity is a broad concept. On the individual level it includes not only the quality of food and food shortage but also physical, social and economic access to food as well as food preferences. Hunger, on the other hand, is the direct feeling of the uneasy or painful sensation of not having enough to eat. Hunger is, therefore, regarded as a more severe form of food insecurity (27). Earlier validation work of the HBSC item of food insecurity found that Greenlandic school children connected the item to “being hungry in the daily life” and “wanting to eat”, but not to wanting to lose weight. The question measures the children's own perception of the home environment and it has not been validated against de facto food availability in their home.

Socio-demographic risk factors to food insecurity were revealed. Most of these were the same as those found previously in literature from Western countries. As in earlier studies, boys in the present study were found to be at an increased risk of food insecurity (23,28). As reported before (7,13,15–17) children living in villages and children from homes with a low family affluence were at risk of food insecurity when analysed by the chi-squared test. However, in the logistic regression model, type of habitation was not found to be significant. This can be explained by, in the case of Greenland, the high correlation between low family affluence and location where families in more remote areas generally have a lower family affluence.

The youngest children reported more frequently having experienced food insecurity, while previous studies have found that young children generally are protected from disrupted eating patterns and reduced food intake at much greater levels of food insecurity in the family than are older teenaged children (29). According to mothers’ narratives, when food becomes scarce, the mother employs a sequence of strategies to manage increasingly severe situations with an overall function of protecting children from hunger except in the most extreme situations (30). The youngest school children in this study reported the highest prevalence of food shortage, indicating that they are too old to be protected by their caregivers but still too young to find other sources to cover their needs. This is consistent with data from interviews which have shown that older teenaged children in insecure families’ express more ways to cope with food insecurity at home than the younger children and how they help to protect their younger siblings from the effect of a food shortage at home (19).

Globally, as well as among Arctic countries, low family affluence or poverty is the single most common cause of food insecurity (2,7,16,17,31,32). Still, food insecurity is to a lesser extent also seen in high affluence families. Greenlandic children themselves pointed to food insecurity being connected to child neglect, and being more common in children living in villages, among homeless people and in children whose parents had spent the money buying other things. This indicates that the ability to acquire food may not be converted into actual food acquisition in all families, and that other causes of food insecurity are relevant for examination. Previous studies have shown many risk factors that increase the risk of food insecurity among children. Generally, most of them are closely associated to poverty, such as minority or immigrant status, low level of educational achievement for the parents, lone parent families, female-headed households and multiple-child households (2,6,16,17,29). However, as indicated by the children themselves, another relevant causal factor might be family management. A study among Irish and Canadian school children found a remarkable consistency in observed relationships with food insecurity to family relations and family management: the highest odds of food poverty was found among children with poor communication with parents, high levels of television viewing, not eating dinner on weekdays, less than weekly tooth-brushing and risky behavior such as non-use of car seatbelts (28). Neglectful and chaotic households have also been associated with parental factors linked to food insecurity, including poor parental mental health, substance abuse, cognitive impairment and limited social support (15).

Perceived health, physical health complaints and medicine use were chosen as outcomes because they were regarded as mirroring 3 different but interrelated health effects. Other studies have concluded that perceived health and health complains in children and adolescents are both signs of physical disease and also a reaction to stressful life events (28,32). Medicine use was chosen as a proxy for health because other studies have shown that medicine use has a strong relation with perceived vulnerability and severity of health problems, furthermore medicine use has been associated with other health risk behaviors and poor self-rated health (33,34). As prescribed medicine is a public expense medicine, use in Greenlandic children is not related to purchasing power within the family. Experiencing food insecurity at home was significantly associated with poorer health effects for all 3 proxy outcomes. The logistic regression showed – both for crude rates and rates controlled for age, gender and FAS – poorer health effects when reporting food insecurity. The association was strongest for self-rated health (OR 1.6) and medicine use (OR 1.8).

The health effects of food insecurity were different for different age groups. The oldest children had the lowest proportion reporting food security and also experienced less negative health effects compared with the younger children. For self-rated health and medicine use, the 11–12 year olds and the 13–14 year olds had higher odds for negative health effects than the oldest children. While the 11–12 year olds reporting food insecurity had doubled their risk for poor or fair health (OR 2.0) and the OR in the 13–14 year-olds was 1.8, the risk was not increased among the oldest children. The risk for medicine use the past month was doubled in both the 11–12 year-olds and in the 13–14 year olds with OR 2.1 and OR 2.0 respectively, but only marginally increased in the oldest children (OR 1.5) (p=0.05). Physical symptoms were only related to food insecurity in the 13–14 year-olds with OR 1.5 (p=0.03) but not in the youngest and oldest children. These results suggest that children in different age groups experience partly different health effects of food insecurity.

### Limitation of the study

The study is cross-sectional and therefore gives no possibility to analyse causality between food insecurity and health effects, especially bearing in mind that a reverse causation in this research area is most likely. Poverty has a major impact on health and perception of health, and due to the close relationship between food insecurity and poverty, causal relations to food insecurity alone are difficult to determine.

There are methodological challenges in comparing findings on food insecurity across countries in the Arctic region. A study using a modification of the USDA measure found that 9% of adults in a small town in north-west Greenland experienced food insecurity, while the same measure found 64 and 83% respectively in 2 Canadian Inuit villages (35). It is most likely that large differences in access to food exist between Arctic countries and that these differences are related to differences in societal organisation and in welfare policies, including governmental subsidisation of basic foods and transport. Still, an inconsistency exists between child and adult reports that nearly the same proportion of children report food poverty in the home in northern Canada and Greenland (23) while at the same time large differences between adult reports on food insecurity have been found with another measure. This calls for further investigations.

## Conclusion

The finding of an association between reporting food insecurity as measured by having experience going to bed or to school hungry because there were no food at home and self-rated health, physical symptoms and medicine use is concerning. Poor health in childhood might have long-term consequences which may continue throughout adulthood (32). The frequent occurrence of food insecurity should, therefore, be seen as an important public health issue. Structural interventions aiming to reduce the occurrence of food insecurity in schoolchildren such as subsidising school meals have already been done in order to prevent immediate adverse effect, for example malnutrition, poor concentration and poor school performance. However, findings suggest that characteristics of parents and households also might contribute to determine whether children's households become food insecure (15,28). If cognitive, behavioral and emotional problems among food-insecure children share a common cause with food insecurity, interventions addressing children's food situations will fail to fully ameliorate poor developmental outcomes. Thus, a variety of social, economic, political and environmental factors must be considered when addressing food insecurity through policy instruments (15).
